# Numerical Modelling of the Dynamic Voltage in HTS Materials under the Action of DC Transport Currents and Different Oscillating Magnetic Fields

**DOI:** 10.3390/ma15030795

**Published:** 2022-01-21

**Authors:** Boyang Shen, Xiaoyuan Chen, Lin Fu, Luning Hao, Tim Coombs

**Affiliations:** 1 Department of Engineering, University of Cambridge, Cambridge CB3 0FA, UK; bs506@cam.ac.uk (B.S.); lin.fu.2017@outlook.com (L.F.); tac1000@cam.ac.uk (T.C.); 2 Clare Hall, University of Cambridge, Cambridge CB3 9AL, UK; 3 School of Engineering, Sichuan Normal University, Chengdu 610101, China; chenxy@sicnu.edu.cn; 4 VLSI Research Europe, Cambridge CB24 6WZ, UK

**Keywords:** high-temperature superconductor (HTS), HTS tape, dynamic voltage, AC magnetic field, oscillating signals, finite-element method (FEM)

## Abstract

The dynamic voltage is a unique phenomenon of superconducting materials. It arises when the superconductor is carrying a DC transport current and spontaneously in subject to an AC magnetic field. This study excavates the aspects that previous studies have not comprehensively investigated: the dynamic voltage in a DC-carrying superconducting tape exposed to different oscillating AC magnetic fields. First, the fundamental physics of dynamic voltage/flux of superconductors is reviewed and further analysed in detail. We used the superconducting modelling method using the ***H***-formulation merged into the finite-element method (FEM) software, to re-produce the typical dynamic voltage behaviour of a superconducting tape. The modelling was verified by both the analytical and experimental results, in order to precisely prove the reliability of the modelling. Afterwards, the modelling was performed for a DC-carrying superconducting tape under four different oscillating magnetic fields (sine, triangle, sawtooth and square), and their corresponding dynamic voltages and energy losses were analysed and compared. Results show the sinusoidal magnetic field can lead to the optimal combination of reasonable dynamic voltage but relatively lower loss, which is suitable for those superconducting applications requiring dynamic voltage as the energy source, e.g., flux pumps. This article presents novel investigation and analysis of the dynamic voltage in superconducting materials, and both the methodology and results can provide useful information for the future design/analysis of superconducting applications with DC transport currents and AC magnetic fields.

## 1. Introduction

Since the superconducting phenomenon was discovered by Kamerlingh Onnes in 1911, superconducting materials have evolved from the Type-I superconductors (many of them are pure metals), to the Type-II superconductors (e.g., oxides and ceramics) [1]. This evolution also promoted the superconducting materials to operate at a relatively higher temperature, from 4.2 K up to over 100 K under atmospheric pressure. Later on, scientists defined the critical temperature (the transition temperature from superconducting state to normal state) as 30 K, to distinguish the low-temperature superconductor (LTS) and high-temperature superconductor (HTS) [2]. The practical superconductors for engineering can be made in the forms of bulks or wires (tapes) [3,4]. For commercial superconducting wires, based on different manufacture processes, there are two generations of superconductors: 1st generation (1G) superconducting wires use the powder-in-tube process, such as the Bi_2_Sr_2_CaCu_2_O_8_ (BSCCO-2212) and Bi_2_Sr_2_Ca_2_Cu_3_O_10_ (BSCCO-2223); 2nd generation (2G) superconducting wires use the coated conductor method to deposit the rare-earth-barium-copper-oxide (REBa_2_Cu_3_O_7_, RE: rare earth [5]) on flexible metal tapes coated by multiple buffering layers [6].

For all these types of superconductors, they have the fascinating advantage of zero-resistance characteristics. However, the zero-resistance behaviour has several constraints: the critical current *I*_c_, critical magnetic field (intensity) *H*_c_ and critical temperature *T*_c_, and these constrains are variables which are dependent on each other [1]. If the superconductor cannot operate within these constraints, the zero-resistance state will be violated. Additionally, the superconductors are lossless with the DC conditions (e.g., DC transport current and DC magnetic field), but superconductors suffer losses when they are carrying AC transports and/or exposed to AC magnetic field [7,8]. These losses can be classified as the ‘AC loss’, which can be instantly converted to the heat dissipation and cause burdens and problems for the cryogenic superconducting system [9].

Another combination for superconductors carrying DC currents and in the presence of AC magnetic fields can also cause AC losses. This is a unique phenomenon of superconductors that a DC voltage is induced (generally defined as the dynamic voltage), and along with the DC transport current a virtual DC resistance can be seen (generally called the dynamic resistance) [10,11,12]. This phenomenon is relevant to superconducting applications such as the nuclear magnetic resonance (NMR) magnets, and superconducting synchronous machines. The dynamic voltage/flux phenomenon is crucial for understanding the mechanism of rectifier-transformer flux pumps [13]. Therefore, the investigations of dynamic voltage/resistance become important for the design of these superconducting applications with high-performance.

The superconducting dynamic voltage/resistance has been analytically studied [10,11,12], and can also be investigated by experiments [14,15,16], and modellings [17,18,19]. However, previous studies only used the sinusoidal signal (or mono-type AC signal) as the waveform of the AC magnetic field, but the systematic investigation of dynamic voltage/resistance using other types of oscillating AC fields is still missing. In addition, studying superconductors under different oscillations could be useful as the parameters and states can change in the oscillations, e.g., the boundary of the YBCO/YMnO_3_ interfaces [20], and there are systems that show superconductivity suppression with RE = Pr substitution [21].

In this article, the modelling was based on the professional superconducting formulation using the finite-element method (FEM). The modelling was verified by agreeing with the analytical characteristics and experiment. Later, a DC-carrying superconducting tape under four different oscillating magnetic fields (sine, triangle, sawtooth and square) was modelled, and their corresponding dynamic voltages and energy losses were analysed and compared. Further study was carried out to find the optimal case which was sensible for sufficient dynamic voltage and relatively low total loss. The novel investigation and analysis of the DC-carrying superconducting tape with different oscillating magnetic fields are useful to provide guidelines for the dynamic voltage/resistance studies, and predict complex situations for relevant superconducting applications.

## 2. Basic Physics of Dynamic Voltage

**Figure 1:**
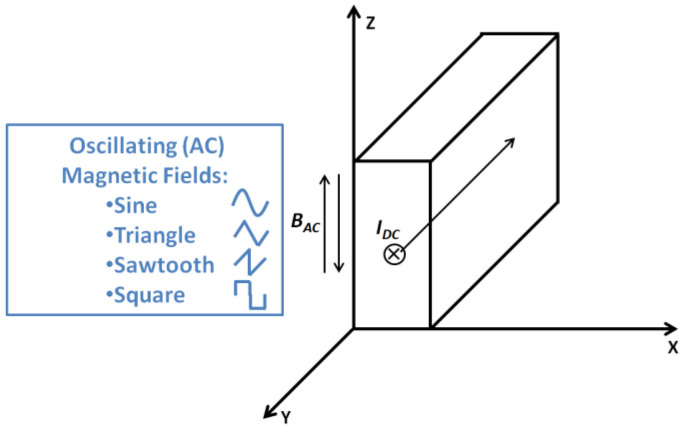
Bean model for a superconducting slab in the presence of an AC magnetic field in the z direction with infinite length in the y-z plane, with the add-ups of a DC transport current in the y direction and four different oscillating magnetic fields (sine, triangle, sawtooth and square).

In order to understand the basic physics of superconducting dynamic voltage/resistance, it is necessary to understand some basics of superconductivity. The physics of superconductivity is complex, which includes the electromagnetics, thermodynamics, materials and quantum mechanics. Fortunately, there are several analytical models which are able to offer convenient approaches to predict the physical phenomenon of superconductivity. One of the most symbolic and widely used analytical models is the Bean Model [22,23], as shown in Figure 1.

The original Bean model (does not include the DC transport current in the y direction) predicts a superconducting slab in the presence of an AC magnetic field in the z direction with infinite length in the y-z plane. Therefore, the surface current in the x direction is negligible compared to the current in the y direction, and assumption is made that induced currents flow only in the y direction (two-way, ‘+’ or ‘−’).

**Figure 2:**
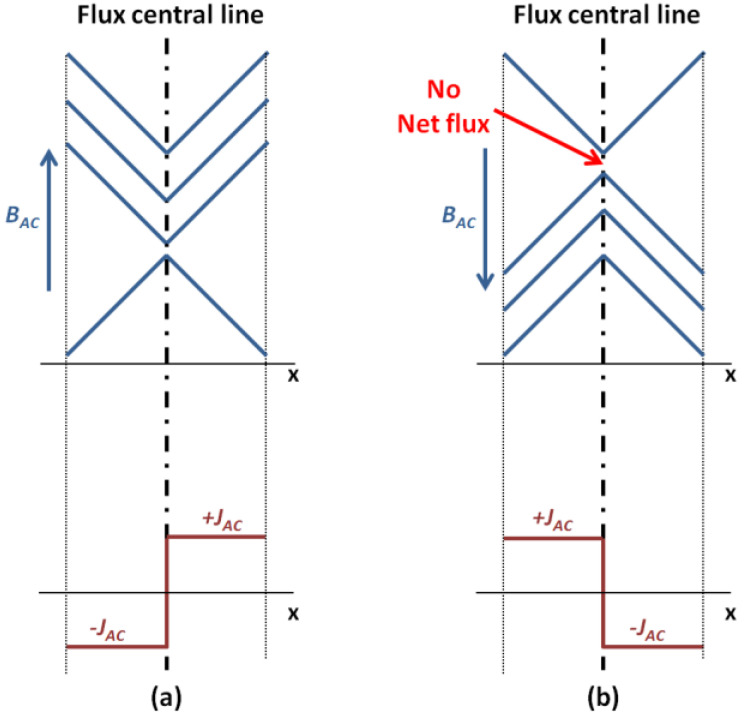

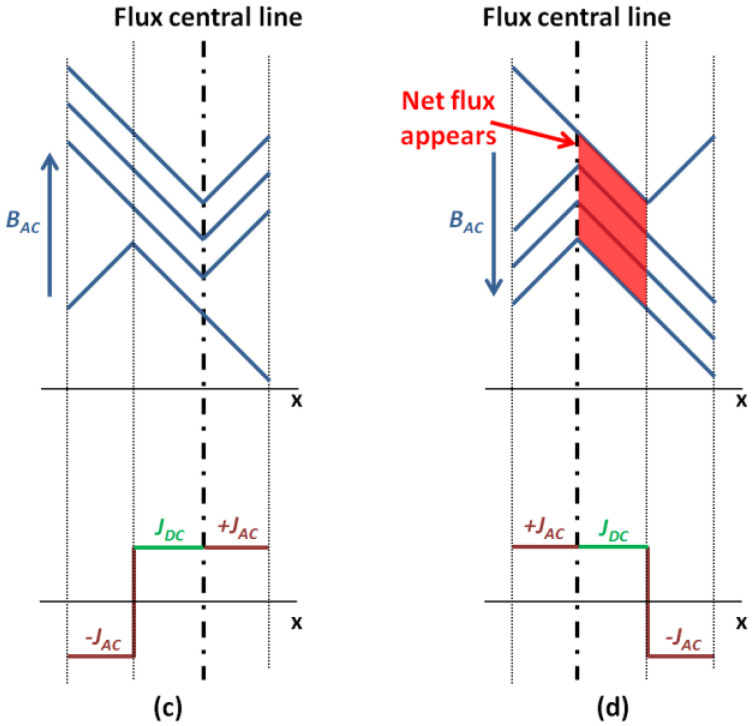
Magnetic field and current profiles of the superconducting slab in the presence of an AC magnetic field in the z direction: (**a**,**b**) the original Bean model (does not include the DC transport current) with no net flux, (**c**,**d**) Bean model with the add-up of a DC transport current in the y direction and the appearance of a net flux in the centre.

For the Bean model without DC transport current in the y direction in Figure 2a,b, when the magnetic field *B_AC_* increases monotonically (in the ‘+’ cycle), screening currents *J_AC_* are induced at the surface of the slab and gradually penetrate into the slab interior. *B_AC_* reaches its peak value of full penetration (*B_P1_*), and both the magnetic field and screening currents *J_AC_* occupy the entire cross-section in the x direction (either −*J_AC_* or +*J_AC_*, in Figure 2a). The slope is the derivative with respect to the magnetic field inside the slab, and the value of screening currents *J_AC_* is always identical to the critical current density:

(1) $$J_{AC}=\pm J_{c}=\frac{\partial \left(B_{AC}/\mu_{0}\mu_{r}\right)}{\partial x}$$

The magnetic field and current density profiles are completely reversed (either +*J_AC_* or −*J_AC_*, in Figure 2b), when the magnetic field *B_AC_* decreases by 2 × *B_P1_* of its peak value (in the ‘−’ cycle). After the whole cycle completes, although the magnetic field profiles has changed for the time being, there is no movement of the flux central line, and there is no net flux induced.

The situation changes when the Bean model is with the add-up of a DC transport current in the y direction. In the ‘+’ cycle, when the magnetic field *B_AC_* increases to reach the peak value of full penetration *B_P2_* (this time *B_P2_* is smaller than *B_P1_*, due to the effect of DC transport current), the entire cross-section in the x direction is occupied by three current components (−*J_AC_*, *J_DC_* and +*J_AC_*, in Figure 2c), and the flux central line moves right to the boundary between *J_DC_* and +*J_AC_*. Similarly, in the ‘−’ cycle, when the magnetic field *B_AC_* decreases by 2 × *B_P2_*, three current components occupy the entire cross-section in the x direction and appear in the order of (+*J_AC_*, *J_DC_* and −*J_AC_*, in Figure 2d), and the flux central line also moves left to the boundary between +*J_AC_* and *J_DC_*. During the whole cycle, the position of flux central line moves to the right edge and then to the left edge, resulting in a net flux Δ*ϕ* flowing across the superconductor:

(2) $$\Delta \phi =\frac{2alI_{DC}}{I_{c}}\left(B_{AC}-B_{AC,th}\right)$$

where *a* and *l* are the width and length (x direction and y direction) of the superconducting slab, *I_c_* is the critical current, *I_DC_* is the DC transport current, *B_AC,th_* is the threshold field for the full penetration. According to the Faraday’s law, this net flux is able to induce a DC voltage that has the same direction as the DC transport current in the superconductor, which is generally called the dynamic voltage:

(3) $$V_{dyn}=\frac{d\phi }{dt}=\frac{2alI_{DC}}{TI_{c}}\left(B_{AC}-B_{AC,th}\right)$$

where *T* is the period of the cycle.

## 3. Modelling Method

The Bean model is a kind of critical state model for superconductors. The ***H***-formulation uses the *E-J* power relation to present the superconducting behaviour that is close to critical state models and even closer to the real superconducting behaviour of HTS. Therefore, the ***H***-formulation was chosen to simulate the dynamic voltage phenomenon of HTS tapes. 

The comprehensive derivation of ***H***-formulation can be found in our previous study [24]. The key governing equation of ***H***-formulation is:

(4) $$\frac{\partial \left(\mu_{0}\mu_{r}H\right)}{\partial t}+\nabla \times \left(\rho \nabla \times H\right)=0$$

where the ***H*** is the magnetic field intensity, *μ_r_* is the relative permeability and *μ*_0_ is the free space permeability.

As the critical current of HTS ReBCO tapes has strong dependence with the magnetic field, an anisotropic field-dependent critical current model was also implemented into the ***H***-formulation:

(5) $$J_{c}\left(B\right)=\frac{J_{c0}}{{\left(1+\frac{\sqrt{{\left(kB_{para}\right)}^{2}+B_{perp}{}^{2}}}{B_{c}}\right)}^{b}}$$

where ***B_perp_*** and ***B_para_*** are the perpendicular and parallel components of magnetic field. Detailed parameters of the modelling can be found in Table 1. Equations (4) and (5), and a full collection of the electromagnetic equations (including the Maxwell’s equations) were merged into the FEM software COMSOL to model the superconducting dynamic voltage phenomenon. The mesh for the superconducting tape domain used the ‘mapped mesh’, and the mesh for the air domain used the ‘free triangular mesh’.

The superconducting tapes for the modelling are the HTS REBCO tapes, with width 4 mm or 6 mm, with the critical current 100 A or 150 A. As the superconducting dynamic voltage phenomenon only appears in the superconducting materials, so in this study other metal and buffer layers were ignored, and only the superconducting layer (thickness 1 μm) was studied in detail.

**Figure 3:**
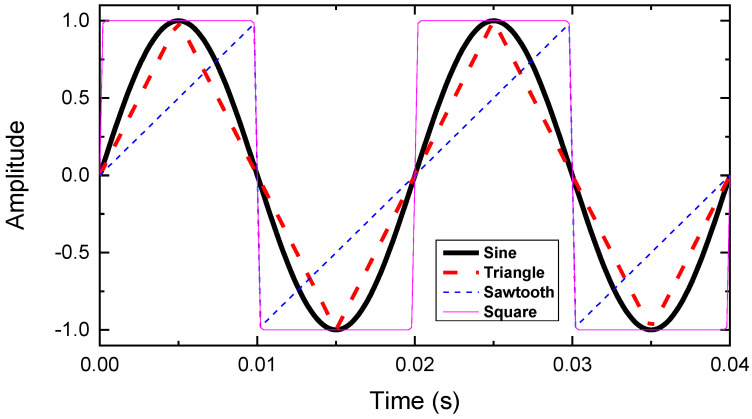
Four oscillating AC signals applied for the magnetic field subject to the HTS tape: sine, triangle, sawtooth and square (modelling signals).

Figure 3 shows four oscillating AC signals applied for the magnetic field subject to the HTS tape: sine, triangle, sawtooth and square (modelling signals). These four signals were with unit magnitude and 50 Hz frequency, and they varied based on the requirements of different cases. In order to make fair comparisons, for both the sawtooth and square waves, their rising edge and falling edge (transition zones) were smoothed to 0.0002 s for 50 Hz.

The 1st step to verify the reliability of modelling method was to re-produce the current density pattern of original Bean model and the effect with the adding-up DC transport current shown in Figure 2.

**Figure 4:**
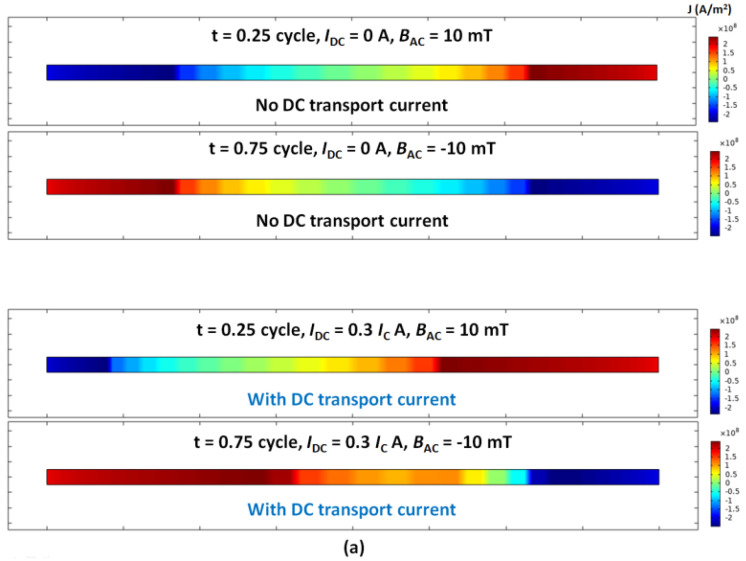

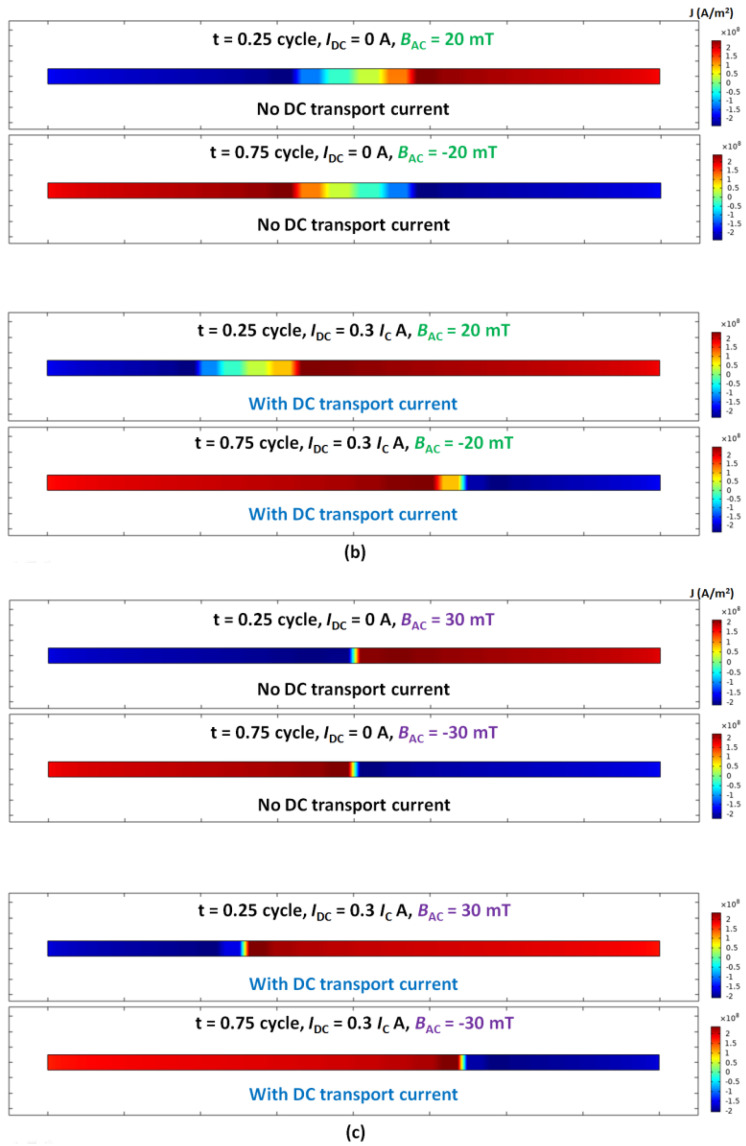
‘No DC transport current’ vs. ‘with DC transport current’: current density distribution of an HTS tape (4 mm width and 1 μm thickness, but here thickness was visually expended × 100 times for better observation), at the time point t = 0.25 cycle and t = 0.75 cycle, with a sinusoidal AC magnetic field perpendicular subject to the HTS tape, with the peak values (**a**) 10 mT, (**b**) 20 mT and (**c**) 30 mT.

Figure 4 shows the comparison of ‘no DC transport current’ vs. ‘with DC transport current’: the current density distribution of an HTS tape (4 mm width and 1 μm thickness, but here thickness was visually expended × 100 times for better observation), at the time point t = 0.25 cycle and t = 0.75 cycle, with a sinusoidal AC magnetic field perpendicularly subject to the HTS tape, with the peak values (a) 10 mT, (b) 20 mT and (c) 30 mT.

For the sinusoidal AC magnetic field, the ***B****_AC_* reached the positive maximum at t = 0.25 cycle, and ***B****_AC_* reached the negative maximum at t = 0.75 cycle. As shown in Figure 4a, with ***B****_AC_* peak value 10 mT but no DC transport current, the ‘+’ and ‘−’ current density distributed limitedly over the two end parts of the HTS tape, and there was a large void zone (no/small current) on the central part of the HTS tape, which matched the typical Bean model at the starting point of increasing the magnetic field. With a DC transport current of 0.3 *I*_c_, the apparent void zone still existed, but was offset either left or right, which also matched the typical analytical solution.

Figure 4b presents the current density distribution of the HTS tape exposed to a perpendicular ***B****_AC_* with peak value 20 mT. The patterns of current distribution were similar to those in Figure 4a, but the void zone became much smaller, which implies the magnetic field was in the intermediate increasing stage, but had not reached the peak value of full penetration.

In Figure 4c, the HTS tape was exposed to a perpendicular ***B****_AC_* with peak value 30 mT. This time, with no DC transport current, the ‘+’ and ‘−’ current density was equally distributed throughout the entire HTS tape, and the void zone almost disappeared, which well matched the typical Bean model shown in Figure 2a,b. This also implies the magnetic field at 30 mT could be close to full penetration field of the HTS tape. If having a DC transport current of 0.3 *I*_c_, the ‘+’ and ‘−’ current density still distributed throughout the whole HTS tape, but the boundary between the ‘+’ and ‘−’ current density was offset either to left or right, whose phenomenon agreed well with the analytical solution shown in Figure 2c,d.

**Figure 5:**
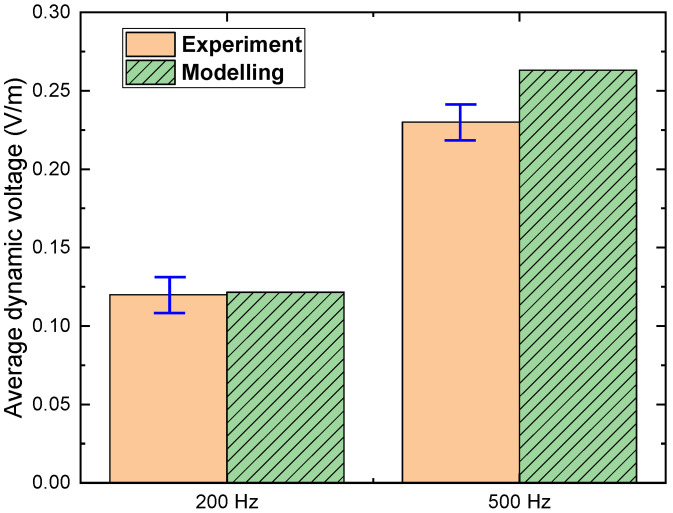
Experiment vs modelling: the average dynamic voltage of a 6 mm HTS tape (*I*_c_ = 135 A), carrying a 80 A DC transport current and in the presence of the perpendicular AC magnetic field at 100 mT with the frequencies at 200 Hz and 500 Hz.

After successfully proving the modelling method was able to re-produce the analytical models, further comparisons were carried out between the modelling and experiment. The experiment was performed by [14], using a 6 mm HTS tape (*I*_c_ = 135 A), carrying 80 A DC transport current and in the presence of the perpendicular AC magnetic field at 100 mT with the frequencies at 200 Hz and 500 Hz (Figure 6a in [14]). The experimental data was estimated from that figure and possible error margins were added, as shown in Figure 5. A model of the HTS tape that was same as the experiment was built, and simulated with same conditions.

Figure 5 shows the comparison of experiment vs modelling: the average dynamic voltage of a 6 mm HTS tape with a DC transport current 80 A and AC magnetic field 100 mT, with two cases (frequencies at 200 Hz and 500 Hz). It can be seen that for the 200 Hz case, the modelling average dynamic voltage matched well with the experimental results, but for the 500 Hz case, the difference between experiment and simulation became slightly higher but still in the acceptable region (more accurate for frequency below 500 Hz). The differences between modelling and experimental results could possibly be due to the ferromagnetic material in the experiment diverted some of the magnetic field, but the modelling used the ideal magnetic field. To summarise, the modelling method shows good agreement with both the experiment and analytical solutions regarding the superconducting dynamic voltage.

## 4. Dynamic Voltages under 4 Oscillating AC Magnetic Fields

After confirming the reliability of superconducting dynamic voltage modelling, more in-depth simulations were performed for a 4 mm DC-carrying HTS tape under four different oscillating AC magnetic fields (sine, triangle, sawtooth and square).

**Figure 6:**
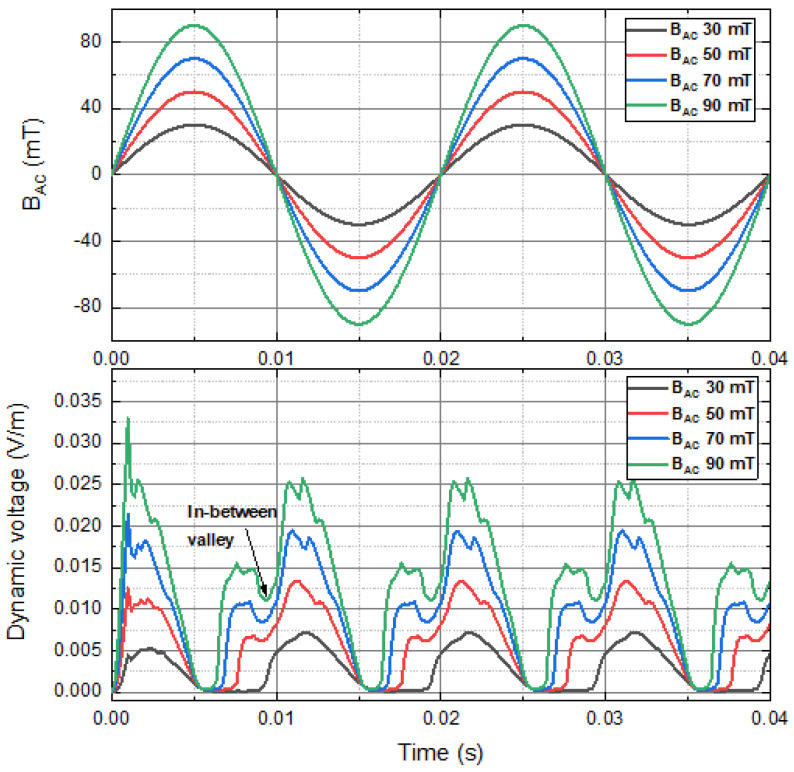
Instantaneous dynamic voltage of a 4 mm HTS tape, carrying a 30 A DC transport current and in the presence of the perpendicular sinusoidal AC magnetic field at 50 Hz, with the peak magnitude 30 mT, 50 mT, 70 mT and 90 mT.

Figure 6 shows the instantaneous dynamic voltage of a 4 mm HTS tape, carrying a 30 A DC transport current and in the presence of the perpendicular sinusoidal AC magnetic field at 50 Hz, with the peak magnitude 30 mT, 50 mT, 70 mT and 90 mT. Results show that the frequency of the instantaneous dynamic voltage was approximately two times that of the frequency of the applied sinusoidal AC magnetic field. The minimum instantaneous dynamic voltage occurred around the sinusoidal AC magnetic field and reached the maximum or minimum, where the time derivative of the magnetic field was zero. The maximum instantaneous dynamic voltage (around 0.025 V/m in the steady-state) happened at each time after the magnetic field just passed through the ‘0’ point. It can be found that some ‘in-between valleys’ occurred just before the magnetic field passing through the ‘0’ point (e.g., around t = 0.009 s). This phenomenon did not happen with the 30 mT sinusoidal magnetic field, but was obvious with the magnetic fields with higher amplitudes. The possible reason could be the magnetisation effects and the interaction between residual current and newly induced current were more significant with the magnetic fields with higher amplitudes.

**Figure 7:**
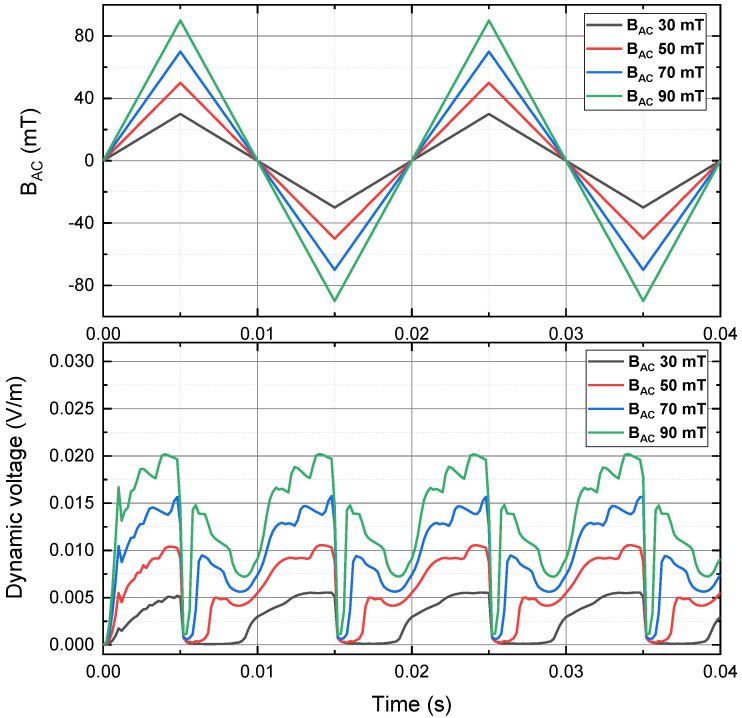
Instantaneous dynamic voltage of a 4 mm HTS tape, carrying a 30 A DC transport current and in the presence of the perpendicular triangle AC magnetic field at 50 Hz, with the peak magnitude 30 mT, 50 mT, 70 mT and 90 mT.

As shown in Figure 7, the instantaneous dynamic voltage of a 4 mm HTS tape is plotted. It was transporting a 30 A DC transport current and in the presence of the perpendicular triangle AC magnetic field at 50 Hz, with the peak magnitude 30 mT, 50 mT, 70 mT and 90 mT. The overall instantaneous dynamic voltage pattern generated by the triangle AC magnetic field was similar to that generated by the sinusoidal AC magnetic field. Compared to the sinusoidal case, there were several differences. The rising edge and falling edge of the instantaneous dynamic voltage with the triangle case were faster than those with the sinusoidal case, which could be due to the time derivative of the sinusoidal wave was mild around peaks, but the time derivative of the triangle wave always had the same magnitude. The maximum instantaneous dynamic voltage with the triangle case (around 0.02 V/m), was smaller than that with sinusoidal case (around 0.025 V/m). The ‘in-between valleys’ phenomenon was more obvious with the triangle case, which could be owing to the stronger interaction of magnetisation and demagnetisation.

**Figure 8:**
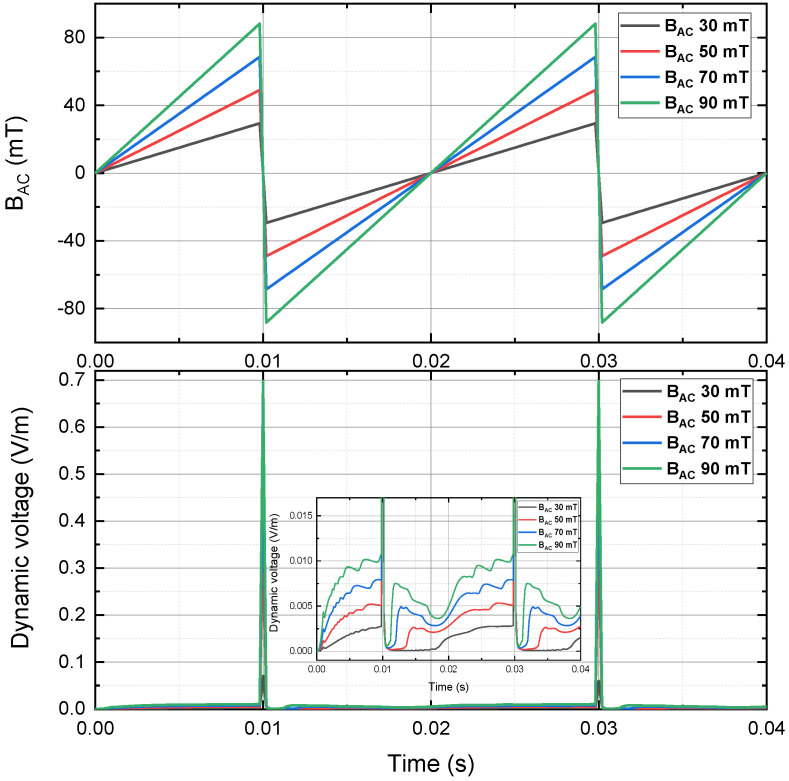
Instantaneous dynamic voltage of a 4 mm HTS tape, carrying a 30 A DC transport current and in the presence of the perpendicular sawtooth AC magnetic field at 50 Hz, with the peak magnitude 30 mT, 50 mT, 70 mT and 90 mT.

Figure 8 depicts the instantaneous dynamic voltage of a 4 mm HTS tape, carrying a 30 A DC transport current and in the presence of the perpendicular sawtooth AC magnetic field at 50 Hz, with the peak magnitude 30 mT, 50 mT, 70 mT and 90 mT. The overall instantaneous dynamic voltage pattern of sawtooth case was much different from the instantaneous dynamic voltage pattern of sinusoidal case and triangle case. The frequency of instantaneous dynamic voltage waveform was the same as the frequency of the sawtooth AC magnetic field, which could be due to the sawtooth signal only had one sharp decreasing part and one relatively mild increasing part in every cycle, but the sinusoidal case and triangle case had 2 up-and-down parts in every cycle. There was a sharp spike of instantaneous dynamic voltage up to 0.7 V/m in each cycle. However, as shown in Figure 8, when the flat waveforms were zoomed in, the maximum instantaneous dynamic voltage in the general region was about 0.01 V/m with the peak magnetic field 90 mT, which was even smaller than the half of the sinusoidal case and triangle case.

**Figure 9:**
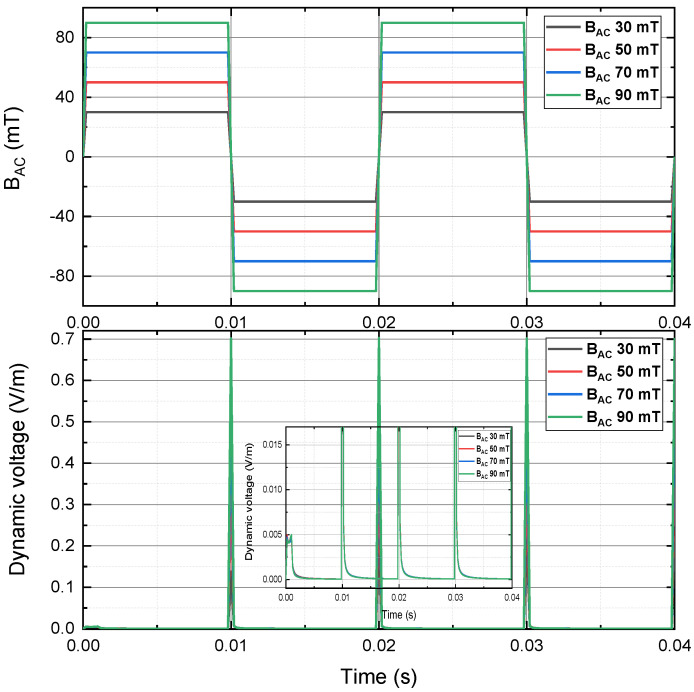
Instantaneous dynamic voltage of a 4 mm HTS tape, carrying a 30 A DC transport current and in the presence of the perpendicular square AC magnetic field at 50 Hz, with the peak magnitude 30 mT, 50 mT, 70 mT and 90 mT.

Figure 9 shows the instantaneous dynamic voltage of a 4 mm HTS tape. It was transporting a 30 A DC transport current and in the presence of the perpendicular square AC magnetic field at 50 Hz, with the peak magnitude 30 mT, 50 mT, 70 mT and 90 mT. Similar to the sawtooth case, the instantaneous dynamic voltage waveforms had sharp spikes, but the instantaneous dynamic voltage frequency of the sawtooth case was two times of the instantaneous dynamic voltage frequency of the sawtooth case. Different from all other three cases (sinusoidal, triangle and sawtooth), although in Figure 9 the flat waveforms were zoomed up, apart from the sharp spikes, the typical dynamic voltage waveforms of other three cases could not be found with the square signal, which could be owing to the square signal kept constant values for most time in every cycle except for the rising and falling edges.

For some superconducting applications like the rectifier-transformer flux pump, the dynamic voltage generated from the superconducting bridge is the energy source for charging the load. However, as shown in Equation (6), there are magnetisation losses in the superconducting materials that account for the total energy supplied by the system power sources. Therefore, it is meaningful to study the composition of dynamic voltage energy within the total energy.

(6) $$Q_{total}=Q_{dynamic}+Q_{magnetisation}$$

**Figure 10:**
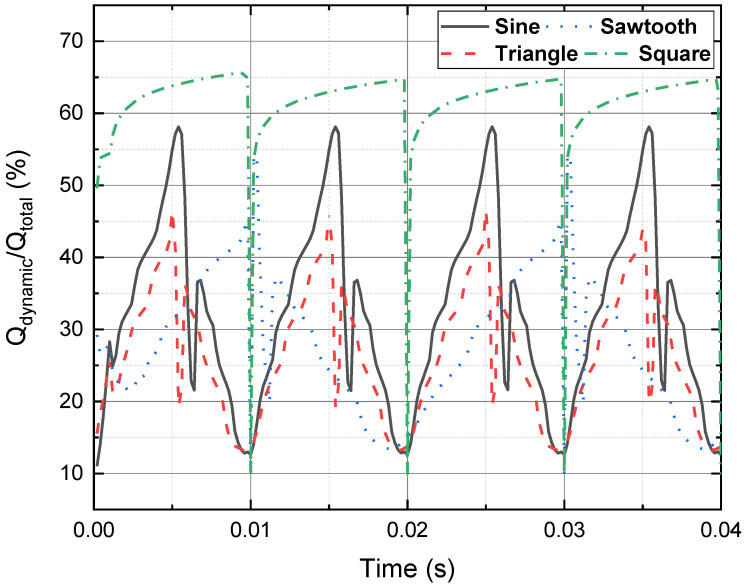
Ratios of *Q_dynamic_*/*Q_total_* of a 4 mm HTS tape, carrying a 30 A DC transport current and in the presence of four different perpendicular AC magnetic fields (sinusoidal, triangle, sawtooth and square) at 50 Hz, with the peak magnitude 90 mT.

**Figure 11:**
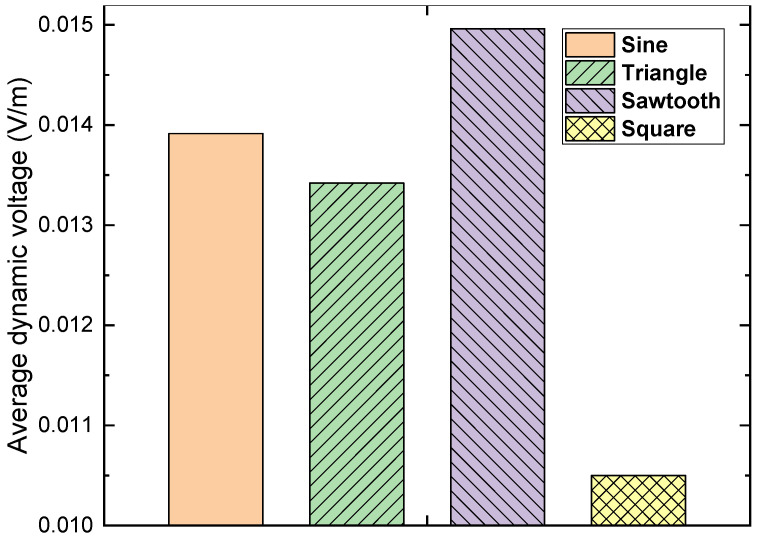
Average dynamic of a 4 mm HTS tape, carrying a 30 A DC transport current and in the presence of four different perpendicular AC magnetic fields (sinusoidal, triangle, sawtooth and square) at 50 Hz, with the peak magnitude 90 mT.

Figure 10 presents the ratios of *Q_dynamic_*/*Q_total_* of a 4 mm HTS tape, carrying a 30 A DC transport current, and in the presence of four different perpendicular AC magnetic fields (sinusoidal, triangle, sawtooth and square) at 50 Hz, with the peak magnitude 90 mT. It can be found the *Q_dynamic_*/*Q_total_* with the sinusoidal magnetic field was higher than the *Q_dynamic_*/*Q_total_* of triangle and sawtooth magnetic fields for most of the time. The *Q_dynamic_*/*Q_total_* with the square magnetic field maintained the highest over the *Q_dynamic_*/*Q_total_* with other three magnetic fields. However, it should be noted that although the ratio *Q_dynamic_*/*Q_total_* with square case was high, the real value of the dynamic voltage was still in a low level. Figure 11 shows the average dynamic of a 4 mm HTS tape, carrying a 30 A DC transport current, and in the presence of four different perpendicular AC magnetic fields (sinusoidal, triangle, sawtooth and square) at 50 Hz, with the peak magnitude 90 mT.

As shown in Figure 11, the average dynamic voltage with the square magnetic field was much smaller than the average dynamic voltage with the other three cases. Although the average dynamic voltage with the sawtooth magnetic field was slightly higher than the average dynamic voltage with the sinusoidal case, its corresponding magnetisation loss was much higher due to the fast transition region. To summarise, the sinusoidal magnetic field was able to lead to reasonably high dynamic voltage, and also with a sensible *Q_dynamic_*/*Q_total_* ratio, which could be the most suitable choice among the four different magnetic fields to energise relevant superconducting devices.

## 5. Conclusions

This article investigates the dynamic voltage in a DC-carrying superconducting tape exposed to four different oscillating AC magnetic fields (sinusoidal, triangle, sawtooth and square), which has not been comprehensively investigated in the previous studies that merely used the sinusoidal signal or mono-type AC signal. The fundamental mechanism of dynamic voltage/flux of superconductors is reviewed and analysed with details. The superconducting modelling method using the ***H***-formulation with the FEM software has successfully re-produced the Bean model and typical dynamic voltage behaviours of a superconducting tape. Both the experimental results and analytical solutions have verified the reliability of modelling. The modelling has been executed for a DC-carrying superconducting tape under sine, triangle, sawtooth and square magnetic fields, and their corresponding dynamic voltage waveforms were analysed with the consideration of energy losses. Results show the sinusoidal magnetic field could give rise to the proper combination of sensible dynamic voltage but relatively low loss, which reveals the sinusoidal magnetic field is appropriate for those superconducting devices requiring dynamic voltage as the energy source, such as flux pumps. The new investigation and analysis of superconducting dynamic voltage behaviours, as well as the methodology and results, can provide useful information for the future design and operation of superconducting applications with DC transport currents and AC magnetic fields.

## Figures and Tables

**Table 1:** Parameters for the superconducting modelling.

Parameters	Value
Tape width	4 or 6 mm
Superconducting layer thickness	1 μm
*n* (*E-J* power relation index)	25
*μ*_0_	4π × 10^−7^ H/m
*E*_0_	10^−4^ V/m
*J_c_*_0_	2.5 × 10^10^ A/m^2^
*b*	0.6
***B**_c_*	35 mT

## Data Availability

The data presented in this study are available on request from the corresponding author.
